# Cross-sectional associations between accelerometer-measured physical activity and hip bone mineral density: the Tromsø Study 2015-2016

**DOI:** 10.1093/jbmrpl/ziae061

**Published:** 2024-05-06

**Authors:** Saija Mikkilä, Bjørn Helge Handegård, Jonas Johansson, Laila A Hopstock, Roland Van den Tillaar, Nina Emaus, Bente Morseth, Boye Welde

**Affiliations:** School of Sport Sciences, UiT The Arctic University of Norway, 9037 Tromsø, Norway; Department of Community Medicine, UiT The Arctic University of Norway, 9037 Tromsø, Norway; The Regional Centre for Child and Adolescent Mental Health – North, UiT The Arctic University of Norway, 9037 Tromsø, Norway; Department of Community Medicine, UiT The Arctic University of Norway, 9037 Tromsø, Norway; Department of Health and Care Sciences, UiT The Arctic University of Norway, 9037 Tromsø, Norway; Department of Sport Sciences and Physical Education, Nord University, 8049 Bodø, Norway; Department of Health and Care Sciences, UiT The Arctic University of Norway, 9037 Tromsø, Norway; School of Sport Sciences, UiT The Arctic University of Norway, 9037 Tromsø, Norway; School of Sport Sciences, UiT The Arctic University of Norway, 9037 Tromsø, Norway; Division of Mental and Physical Health, Department of Child and Adolescent Health Promotion Services, Norwegian Institute of Public Health, 0404 Oslo, Norway

**Keywords:** DXA, general population studies, exercise, osteoporosis, epidemiology

## Abstract

Positive associations between physical activity and bone health have been found in population-based studies, however, mostly based on self-reported physical activity. Therefore, we investigated the association between accelerometer-measured physical activity, measured in steps per day and minutes of moderate to vigorous physical activity (MVPA) per day, and total hip areal BMD (aBMD) measured by DXA in a general population, utilizing multiple regression models. The study participants, 1560 women and 1177 men aged 40–84 yr, were part of the seventh survey of the Tromsø Study (2015-2016). In both genders, we found a positive association between the number of daily steps and aBMD adjusted for age, BMI, and smoking status (*P* < .001). In women, an increase of 1000 steps per day was associated with 0.005 g/cm^2^ higher aBMD. For men, a polynomial curve indicated a positive association with aBMD up to 5000 steps per day, plateauing between 5000 and 14 000 steps, and then increasing again. Additionally, MVPA duration was positively associated with aBMD in both women (*P* < .001) and men (*P* = .004) when adjusted for age, BMI, and smoking status. Specifically, each 60-min increase in daily MVPA was associated with 0.028 and 0.023 g/cm^2^ higher aBMD in women and men, respectively. Despite positive associations, the clinical impact of physical activity on aBMD in this general population of adults and older adults was relatively small, and a large increase in daily MVPA might not be achievable for most individuals. Therefore, further longitudinal population-based studies incorporating device-based measures of physical activity could add more clarity to these relationships.

## Introduction

Bone mineral density is a strong predictor of hip fracture, the most severe among fragility fractures, which often lead to reduced quality of life, severe morbidity, and increased mortality.^1-4^ Furthermore, hip fractures contribute to substantial economic burden in form of hospitalization and rehabilitation.^3^ Physical inactivity is known to be an important risk factor for bone health^5^ and previous studies suggest that physical activity improves BMD, by mechanically stimulating bone cells and leading to formation and bone gain, and reduces fall incidence, thereby reducing the risk of hip fractures.^5-7^

A recent systematic review of randomized controlled trials with participants aged 65 yr and older shows a small but significant effect of physical activity on BMD,^8^ although it is unclear how generalizable these findings are to the general population. Findings from population-based observational studies can complement intervention studies,^8^ and show positive associations between self-reported physical activity and hip BMD in various populations.^9-12^ However, self-report measures are often more prone to recall and response biases, in comparison with objective measures.^13^ Accelerometers have been available as objective measure of physical activity for decades.^14^ However studies of the association between accelerometer-measured physical activity and BMD are scarce and limited to samples of small size,^15-17^ women only^15^^,^^18^ or children and adolescents.^11^^,^^19-21^ When comparing low duration physical activity, i.e., less than 5 minutes daily, to moderate and vigorous physical activity (MVPA), a positive association between accelerometer-measured high duration activity for at least 20 minutes daily, and hip BMD was observed in a large cohort of women and men aged 50 years and older, but not in those under 50 years. In women, the association was also found when the duration was intermediate.^22^ Based on the same national survey, positive associations between light PA and MVPA, and hip BMD were found also in a large cohort of 23-90+ yr old women and men, respectively.^23^ Furthermore, positive associations between accelerometer-measured MVPA and hip BMD were demonstrated in a large cohort of 70-yr-old men and women,^24^ while in another study only in men 65 yr and older, and not in similar aged women.^25^ These equivocal findings need to be further elucidated including both sexes.

Therefore, the aim of this cross-sectional study was to investigate the associations between accelerometer-measured physical activity, and total hip areal BMD (aBMD) in a large population-based sample of adult and older adult women and men study.

## Materials and methods

### Design, sample, and ethical approval

**Figure 1:**
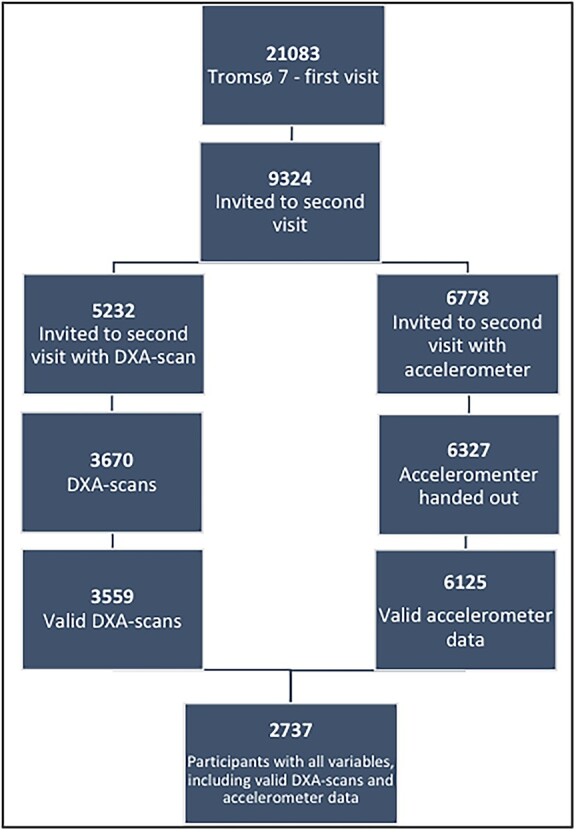
Flowchart illustrating the procedure for selection of participants.

The Tromsø Study is an ongoing population-based study^26^ including 7 surveys to date (1974-2016, Tromsø1–Tromsø7). Consisting of urban and rural living areas, the study is conducted in the municipality of Tromsø, Norway, which is similar to the general Norwegian population according to age^27^ and gender.^27^ The present study includes data from Tromsø7 (2015-2016),^28^ to which all inhabitants ≥40 yr were invited (N = 32 591) to visit 1. Visit 1 included questionnaires, biological sampling, and clinical examinations. A subsample (N = 13 028) consisting of a random sample of 20% aged 40–59 yr and 50% aged 60–84 yr (n = 9925) as well as previous participants attending DXA, echocardiography, and/or eye examinations in Tromsø6 (n = 3103) was pre-marked to visit 2 approximately 3–4 wk later. Visit 2 included extended clinical examinations, including DXA and accelerometer measurements. Invitation to visit 2 required attendance at visit 1. In total 21 083 (participation 65%) attended the first visit (Figure 1). Of these, 9253 were pre-marked for visit 2 invitations. In total, 8346 attended visit 2 (comprising 64% the originally pre-marked visit 2 sample, 90% of those attending visit 1). The present study included 2737 participants; 1560 women and 1177 men aged 40–84 yr who attended both DXA-scanning and wore accelerometers, and with valid data on confounders. All participants provided written informed consent prior to inclusion. Tromsø7 was approved by the Data Inspectorate of Norway and the Regional Committee of Medical and Health Research Ethics, North Norway (2014/940).

### Assessment of physical activity

Objective data on physical activity were assessed by an ActiGraph wGT3X-BT accelerometer (ActiGraph, LLC, Pensacola, United States) and expressed as steps per valid day and as minutes of MVPA per valid day. A valid day comprised the wear time of 4 days, at least 10 h per day. Trained technicians instructed the participants to wear the accelerometer 24 h a day for 8 consecutive days prior to attaching the device to the participants’ right hip at the examination site. The device was programmed to start the data collection at 00:00 the next day and measure continuously for 7 days. Removing the device was advised during water contact, eg when showering / bathing / swimming, and during contact sports. Raw acceleration data were collected with a sampling rate of 100 Hertz. The step count of the accelerometer was derived from the axial plane, based on a manufacturer’s algorithm. The triaxial vector magnitude (VM) counts per minute (CPM) cut-points for different intensities were determined as follows: sedentary behavior: <150, light physical activity: 150–2689, and MVPA: ≥2690 VM CPM. More details of the data processing are described in Sagelv et al.^29^^,^^30^

### Measurement of BMD

Areal BMD was measured using a DXA device (Lunar Prodigy, GE Medical Systems, Madison, WI, United States). All scans were performed according to standard procedures set by GE Medical Systems. The DXA device was calibrated daily using a standard phantom. Trained technicians performed the scanning according to a standardized protocol, and one of them performed quality assessment by visually reviewing each scan of the total sample afterwards. In a validation study, the short-term in vivo precision error for the Lunar Prodigy was 1.7% and 1.2% for the femoral neck and total hip measurements, respectively.^31^ Left total hip scans which include the femoral neck, trochanter, and shaft regions were used for all our analyses.^32^

### Additional measurements

Participants’ height and weight were measured with light clothing and no shoes to nearest centimeter and half-kilogram respectively, using a Jenix DS-102 scale (DongSahn Jenix, Seoul, Korea). Body mass index was calculated from weight and height (kg/m^2^). Smoking (current, previous or never) was self-reported.

### Statistical analyses

Multiple linear and non-linear regression models were used to analyze associations between objectively measured physical activity (per 1000 steps and per minute of MVPA per day) and hip bone aBMD separately for men and women, controlling for BMI, age, and smoking status. Choice of adjustment variables is based on previous literature,^33^ and thus include variables that are commonly known to affect bone health^34^ and available in the Tromsø Study data. Analyses were performed separately for each activity variable. Effect sizes were reported as partial eta squared (η_p_^2^). We assessed the degree of plateauing effect for the number of steps and for the number of daily minutes with moderate and vigorous activity by the curve estimation procedure in SPSS. In the regression analysis the activity variables were centered. We checked the linearity, homogeneity, and normal distribution assumptions, and whether there were influential observations using regression diagnostics (mainly by assessing residual plots and outlier statistics).

We used a significance level of .01 in all tests. SPSS Statistics for Windows v. 28 (IBM Corp. Released 2021. Armonk, NY, United States) was used for the analyses.

## Results

### Sample characteristics

Table 1 displays sample characteristics for women and men separately. Both women and men had an age range of 40–84 yr and a BMI of 13.7–50.6 and 17.0–42.6 kg/m^2^, respectively. The proportion reporting current or previous smoking was larger among men (63.7%) than among women (58.7%). In addition, a majority of men and women (>65%) achieved the WHO’s recommendations for physical activity, ie at least 150–300 min of moderate-intensity aerobic physical activity, or at least 75–150 min of vigorous-intensity aerobic physical activity or an equivalent combination of moderate- and vigorous-intensity activity throughout the week.^35^ (Table 1).

**Table 1:** Sample characteristics per sex. The Tromsø Study 2015-2016.

	**Women (*n* = 1560)**	**Men (*n* = 1177)**
Age (yr)	66.2 (8.7)	66.4 (8.7)
Height (m)	1.63 (0.06)	1.76 (0.07)
Weight (kg)	71.0 (12.7)	85.9 (13.2)
BMI (kg/m^2^)	26.7 (4.7)	27.5 (3.7)
Smoking daily (%) (*n*)		
Never (%) (*n*)	41.3 (645)	36.3 (427)
Current (%) (*n*)	11.5 (179)	9.0 (106)
Previous (%) (*n*)	47.2 (736)	54.7 (644)
Left hip total BMD (g/cm^2^)	0.91 (0.13)	1.04 (0.14)
Accelerometer wear (valid days)	6.8 (0.5)	6.8 (0.5)
Accelerometer wear, time per valid day (h)	17.2 (1.7)	17.2 (2.0)
Steps per valid day^a^ (steps/day)	6840 (2928)	6814 (2865)
Min in MVPA/day^b^^,^^c^	36.7 (27.5)	43.1 (32.1)
MVPA ≥150 min/wk (%) (*n*)	65.3 (1018)	71.1 (838)

Numbers are mean (SD) or percentage (*n*).

a Hecht, 2009.^36^

^b^Moderate and vigorous physical activity minutes per valid day.

c Sasaki^37^ and Kozey-Keadle.^38^

### The degree of plateauing of the effect of objectively measured activity on aBMD

**Figure 2:**
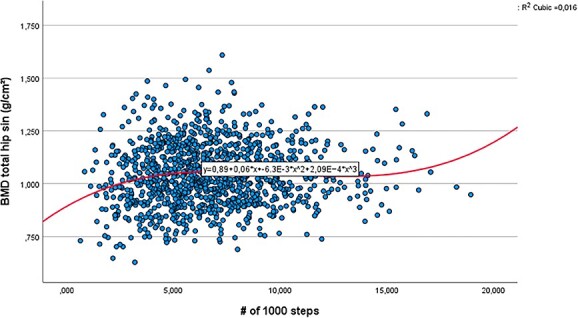
Curve-fit for a cubic model for the association between aBMD and the daily number of steps (in thousands) for men *n* = 1177. The Tromsø Study 2015-2016.

**Figure 3:**
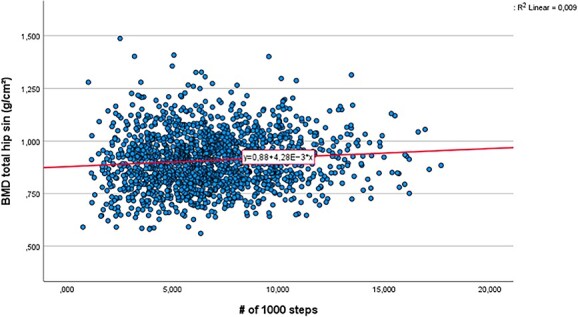
Curve-fit for a linear model for the association between aBMD and the daily number of steps (in thousands) for women *n* = 1560. The Tromsø Study 2015-2016.

We checked whether a linear model was sufficient to study the association between the number of daily steps or minutes MVPA per day and aBMD by testing whether parameters associated with quadratic and cubic terms were significantly different from zero. For men, adding a quadratic and cubic term for the number of daily steps variable improved the fit of the curve, and consequently a quadratic and a cubic term for the steps variable were included in the model (Figure 2). For the variable number of minutes in MVPA, no quadratic or cubic term was significant in the final regression analysis for men. For women, no quadratic or cubic terms were added because these were not significant (Figure 3).

### Association between number of daily steps and aBMD

#### Women

The association between number of daily steps and aBMD for women is shown in Figure 3. Adding number of daily steps to a model containing age, BMI, and smoking status, the proportion of total variance in aBMD (R^2^) increased from 0.264 to 0.274. This small change in explained variance was significantly different from zero (t_(1555)_ = 4.73; *P* < .001; partial regression coefficient = 0.005), ie an increase of 1000 steps was associated with 0.005 g/cm^2^ higher aBMD. To put the association of number of daily steps with aBMD into perspective, we report effect size (partial eta squared, η_p_^2^) for all the independent variables: number of steps: η_p_^2^ = 0.014; BMI: η_p_^2^ = 0.206; age: η_p_^2^ = 0.075; smoking status: η_p_^2^ = 0.006.

#### Men

Because the curve estimation procedure indicated a slightly curved association between the number of daily steps and aBMD, with significant quadratic and cubic terms, the activity variable was represented with a linear, a quadratic, and a cubic term in the model. The polynomial curve fitted shows an initial positive association between aBMD and up to 5000 steps (Figure 2). The curve plateaued between approximately 5000 and 14 000 steps and shows a small positive effect above 14 000 steps. R^2^- increased 0.015 (R^2^ from 0.123 to 0.138) when adding the number of daily steps terms to a model containing the control variables. This increase was significantly different from zero (F_(3, 1169)_ = 6.91; *P* < .001.) Effect sizes for independent variables in the model (η_p_^2^) were: steps: linear term: 0.0005, quadratic term: 0.011, cubic term: 0.008; BMI: 0.088; age: 0.009; smoking status: 0.013.

### Association between minutes MVPA per day on aBMD

#### Women

Adding minutes in MVPA to a model containing age, BMI, and smoking status, R^2^ increased from 0.264 to 0.271, indicating a small, but significant association between minutes in MVPA and aBMD (R^2^ change = .008; *t*_(1555)_ = 4.02; *P* < .001; partial regression coefficient = 0.000462). Each 60-min increase in MVPA was associated with 0.028 g/cm^2^ higher aBMD. Effect sizes for the independent variables in the model (η_p_^2^) were as follows: minutes MVPA: 0.010; BMI: 0.204; age: 0.084; smoking status: 0.006.

The results of the 2 separate analyses; steps and MVPA for women were fairly similar, and the 2 activity variables, steps and MVPA, were highly correlated (r = 0.89).

#### Men

Adding minutes in MVPA to a model containing age, BMI, and smoking status, R^2^ increased from 0.123 to 0.129. The R^2^ change of 0.006 was significantly different from zero (*t* = 2.89; *P* = .004; partial regression coefficient = 0.000381). Each 60-min daily increase in MVPA was associated with 0.023 g/cm^2^ higher aBMD. Effect sizes for independent variables in the model (η_p_^2^) were: MVPA: 0.007; BMI: 0.086; age: 0.013; smoking status: 0.012. As for women, steps and MVPA were highly correlated (r = 0.86).

## Discussion

In this cross-sectional study including 40–84-yr-old women and men from a general population, objectively measured physical activity was positively associated with hip aBMD, although the association is weak. Keeping that in mind, the main findings of this study were: (1) In women and men, the number of daily steps was positively associated with hip aBMD; (2) In men, a slightly curved positive association between the number of daily steps and aBMD was indicated (the curve plateaued between 5000 and 14 000 steps), while in women this association was linear; and (3) In women and men, MVPA was positively associated with hip aBMD. These findings were independent of age, BMI, and smoking status.

The estimated 60-min increase in MVPA per day was associated with 0.023 and 0.028 g/cm^2^ higher aBMD in men and women, respectively. To be noted here is, however, that an increase in physical activity level of this magnitude may be a lot for most individuals. The effect sizes were small, as even as much as 60-min increase in MVPA per day was associated with a small estimated increase in aBMD in both men and women. Yet, even a small effect on BMD may be relevant for fracture risk,^1^ and given that the physical activity levels were measured over a relatively short period of 1 wk and the cross-sectional design of this study, longitudinal studies with objectively measured physical activity are warranted.

While our study has identified a significant and yet small positive association between an increase in MVPA and hip aBMD, it is important to interpret these findings within the context of our study’s cross-sectional design. Despite the inherent limitations in establishing causality, the association we have documented is consistent with a systematic review by Mohebbi et al.,^39^ suggesting a potential causal link between physical activity and BMD. Additionally, research by Soares et al.^40^ supports the notion that an active lifestyle may contribute to a reduced risk of falls among older adults, which is an important consideration for bone health. Since a decrease of 1 SD in BMD at the hip, spine, or wrist is associated with a doubling in fracture risk,^1^ any preventive measure connected to reduction of bone loss is important.

Physical inactivity is known to be an important risk factor for bone health,^5^ often indicated through lower BMD and higher risk of osteoporotic fractures,^1^^,^^41^ Positive associations between physical activity and hip BMD in different populations are well documented in cross-sectional studies^9-12^^,^^21^ although study designs are largely based on self-reported physical activity, like Hauger et al.^10^ who studied associations between self-reported physical activity and hip aBMD using data from Tromsø7, ie the same population as in our analysis. In their study, active women (physical activity level 2–4) had 4.1–10.2% higher aBMD than inactive (physical activity level 1) women. Men at physical activity level 3 had 4.3% higher aBMD compared with level 1, and men >65 yr had 3.1–4.3% (physical activity level 2–3) higher aBMD compared with level 1. Despite measuring physical activity by questionnaire, these findings indicate similar positive associations between physical activity and aBMD as observed in our study. The plateauing of the daily steps seen among men in our study might explain the non-significantly higher aBMD among men in physical activity level 4 compared with level 1 in the study of Hauger et al.,^10^ even though we cannot explicitly point out the plateau on the level 1 to level 4 scale in our analyses.

Few previous studies have investigated objectively measured physical activity and bone health with a reasonably large study sample, which provides statistical power for stratified analyses such as comparing men and women. Similar to the current study, Johansson et al.^24^ found positive associations between MVPA and hip aBMD, although their findings are only generalizable to 70-yr-olds and there were no stratified analyses showing results for women and men separately. In a sample of 2114 women and men aged 23–90+, Chastin et al.^23^ found that time spent in MVPA was associated with higher hip BMD in men. In women, sedentary behavior was negatively and light physical activity positively associated with hip BMD. Interestingly, and in contrast to our findings, Chastin et al.^23^ did not find any associations between MVPA and BMD in women. In a similar study population, intermediate and high durations of MVPA were associated with hip BMD in women aged 50 yr and above, and high duration of MVPA was associated with hip BMD in men aged 50 yr and above.^22^ Furthermore, in a smaller study (*n* = 214), Hind et al.^16^ found higher volumes of light physical activity but not MVPA to be positively associated with BMD. Gaba et al.^15^ and McMillan et al.^17^ found body composition to be a stronger predictor of BMD than physical activity variables, which is also seen in our analyses, showing that BMI together with age and smoking status were stronger predictors for BMD than physical activity. One of the relatively strong effects of BMI on BMD is most likely related to the load placed upon weight-bearing bones, since, in general, greater body weight increases the effects of weight-bearing activity on bone adaptation.^42^ However, physical activity reaches evidence grading A and B, respectively, in the position statement by Weaver et al.^42^ for its effects on bone mass and density, and bone structural outcomes. Since we in our study found BMI (together with age and smoking status) to be a stronger predictor for BMD than physical activity, it is possible that our older subjects may not have engaged in physical activity intense enough to trigger an osteogenic response. It is known that, for example, repetitive low-magnitude loads, which may be a characteristic of the activities chosen by our study participants, are not osteogenic.^42-44^ Also, an animal trial suggests that the osteogenic response is weaker in aging skeleton compared with a young one.^45^ However, according to Weaver et al.,^42^ our understanding of the specific dimensions of physical activity that are osteogenic is incomplete. Hence, further research should focus on what frequency, intensity, time, and type of physical activity are needed to optimize bone structural outcomes in different age segments in men and women.

## Strengths and limitations

To our knowledge, our study is one of the largest studies investigating the association between objective measures of physical activity and BMD in a general population with a wide age range. Due to the cross-sectional design, we cannot establish causal relations. Furthermore, due to fairly slow bone density development, repeated measurements could have strengthened our study by allowing us to study possible changes over time, but no follow-up data with accelerometer-measured physical activity are yet available. Moreover, we did not study type of physical activity, only volume and intensity. Weight-bearing exercises are found to be particularly beneficial,^5^ whereas cycling does not seem to contribute as much to the osteogenic stimulus that is needed for improving bone health.^46^ Activities such as cycling, resistance training, and swimming may be underestimated in our study due to the accelerometer’s reduced validity in measuring those activities.^47^ Therefore, being able to identify the type of physical activity participants engaged in would help us to gain more detailed knowledge on associations between physical activity and BMD. We followed WHO’s recommendations for physical activity, ie MVPA,^35^ and therefore we have chosen not to include light physical activity in our study, which may not imply weight-bearing activities to the same degree as MVPA. Moreover, our analysis did not account for additional confounders such as dietary factors or general health status.

## Conclusion

In this cross-sectional study of women and men from a general population, accelerometer-measured physical activity was positively associated with total hip aBMD, after controlling for age, BMI, and smoking status. Furthermore, the pattern of association varied by sex, as the prediction curve for men plateaued between 5000 and 14 000 steps indicating a curved association. Further longitudinal population-based studies using objective measures of physical activity are warranted to confirm both the magnitude and the direction of these associations, although our findings indicate that maintaining physical activity levels means maintaining bone health in the general population.

## Supplementary Material

supplementary_material_ziae061

## Data Availability

The legal restrictions on data availability are set by the Tromsø Study Data and Publication Committee in order to control for data sharing, including publication of datasets with the potential of reverse identification of de-identified sensitive participant information. The data can however be made available from the Tromsø Study upon application to the Tromsø Study Data and Publication Committee. Contact information: The Tromsø Study, Department of Community Medicine, Faculty of Health Sciences, UiT The Arctic University of Norway; e-mail: tromsous@uit.no

## References

[1] Marshall D , Johnell O, Wedel H. Meta-analysis of how well measures of bone mineral density predict occurrence of osteoporotic fractures. BM*J*. 1996;312(7041):1254–1259. 10.1136/bmj.312.7041.1254 8634613 PMC2351094

[2] Hasserius R , Karlsson MK, Jonsson B, Redlund-Johnell I, Johnell O. Long-term morbidity and mortality after a clinically diagnosed vertebral fracture in the elderly--a 12- and 22-year follow-up of 257 patients. Calcif Tissue In*t*. 2005;76(4):235–242. 10.1007/s00223-004-2222-2 15812579

[3] Cummings SR , Melton LJ. Epidemiology and outcomes of osteoporotic fractures. Lance*t*. 2002;359(9319):1761–1767. 10.1016/S0140-6736(02)08657-9 12049882

[4] Schnell S , Friedman SM, Mendelson DA, Bingham KW, Kates SL. The 1-year mortality of patients treated in a hip fracture program for elders. Geriatr Orthop Surg Rehabi*l*. 2010;1(1):6–14. 10.1177/2151458510378105 23569656 PMC3597289

[5] Kohrt WM , Bloomfield SA, Little KD, Nelson ME, Yingling VR, American College of Sports M. American College of Sports Medicine position stand: physical activity and bone health. Med Sci Sports Exer*c*. 2004;36(11):1985–1996. 10.1249/01.MSS.0000142662.21767.58 15514517

[6] Warburton DE , Nicol CW, Bredin SS. Health benefits of physical activity: the evidence. CMA*J*. 2006;174(6):801–809. 10.1503/cmaj.051351 16534088 PMC1402378

[7] Gregg EW , Pereira MA, Caspersen CJ. Physical activity, falls, and fractures among older adults: a review of the epidemiologic evidence. J Am Geriatr So*c*. 2000;48(8):883–893. 10.1111/j.1532-5415.2000.tb06884.x 10968291

[8] Pinheiro MB , Oliveira J, Bauman A, Fairhall N, Kwok W, Sherrington C. Evidence on physical activity and osteoporosis prevention for people aged 65+ years: a systematic review to inform the WHO guidelines on physical activity and sedentary behaviour. Int J Behav Nutr Phys Ac*t*. 2020;17(1):150. 10.1186/s12966-020-01040-4 33239014 PMC7690138

[9] Sipila S , Tormakangas T, Sillanpaa E, et al. Muscle and bone mass in middle-aged women: role of menopausal status and physical activity. J Cachexia Sarcopenia Muscl*e*. 2020;11(3):698–709. 10.1002/jcsm.12547 32017473 PMC7296268

[10] Hauger AV , Holvik K, Bergland A, et al. Physical capability, physical activity, and their association with femoral bone mineral density in adults aged 40 years and older: the Tromso study 2015-2016. Osteoporos In*t*. 2021;32(10):2083–2094. 10.1007/s00198-021-05949-9 33864108 PMC8510966

[11] Lorentzon M , Mellstrom D, Ohlsson C. Association of amount of physical activity with cortical bone size and trabecular volumetric BMD in young adult men: the GOOD study. J Bone Miner Re*s*. 2005;20(11):1936–1943. 10.1359/JBMR.050709 16234966

[12] Torstveit MK , Sundgot-Borgen J. Low bone mineral density is two to three times more prevalent in non-athletic premenopausal women than in elite athletes: a comprehensive controlled study. Br J Sports Me*d*. 2005;39(5):282–287 discussion -7. 10.1136/bjsm.2004.012781 15849292 PMC1725217

[13] Prince SA , Adamo KB, Hamel ME, Hardt J, Connor Gorber S, Tremblay M. A comparison of direct versus self-report measures for assessing physical activity in adults: a systematic review. Int J Behav Nutr Phys Ac*t*. 2008;5(1):56. 10.1186/1479-5868-5-56 18990237 PMC2588639

[14] Troiano RP , McClain JJ, Brychta RJ, Chen KY. Evolution of accelerometer methods for physical activity research. Br J Sports Me*d*. 2014;48(13):1019–1023. 10.1136/bjsports-2014-093546 24782483 PMC4141534

[15] Gaba A , Kapus O, Pelclova J, Riegerova J. The relationship between accelerometer-determined physical activity (PA) and body composition and bone mineral density (BMD) in postmenopausal women. Arch Gerontol Geriat*r*. 2012;54(3):e315–e321. 10.1016/j.archger.2012.02.001 22405095

[16] Hind K , Hayes L, Basterfield L, Pearce MS, Birrell F. Objectively-measured sedentary time, habitual physical activity and bone strength in adults aged 62 years: the Newcastle Thousand Families Study. J Public Health (Oxf*)*. 2020;42(2):325–332. 10.1093/pubmed/fdz029 31220295

[17] McMillan LB , Aitken D, Ebeling P, Jones G, Scott D. The relationship between objectively assessed physical activity and bone health in older adults differs by sex and is mediated by lean mass. Osteoporos In*t*. 2018;29(6):1379–1388. 10.1007/s00198-018-4446-4 29532131

[18] Sanudo B , de Hoyo M, Del Pozo-Cruz J, et al. A systematic review of the exercise effect on bone health: the importance of assessing mechanical loading in perimenopausal and postmenopausal women. Menopaus*e*. 2017;24(10):1208–1216. 10.1097/GME.0000000000000872 28538603

[19] Janz KF , Letuchy EM, Burns TL, Eichenberger Gilmore JM, Torner JC, Levy SM. Objectively measured physical activity trajectories predict adolescent bone strength: Iowa Bone Development Study. Br J Sports Me*d*. 2014;48(13):1032–1036. 10.1136/bjsports-2014-093574 24837241 PMC4550443

[20] Christoffersen T , Winther A, Nilsen OA, et al. Does the frequency and intensity of physical activity in adolescence have an impact on bone? The Tromso Study, Fit Futures. BMC Sports Sci Med Rehabi*l*. 2015;7(1):26. 10.1186/s13102-015-0020-y 26561526 PMC4641333

[21] Deere K , Sayers A, Rittweger J, Tobias JH. Habitual levels of high, but not moderate or low, impact activity are positively related to hip BMD and geometry: results from a population-based study of adolescents. J Bone Miner Re*s*. 2012;27(9):1887–1895. 10.1002/jbmr.1631 22492557 PMC3465797

[22] Jain RK , Vokes T. Physical activity as measured by accelerometer in NHANES 2005-2006 is associated with better bone density and trabecular bone score in older adults. Arch Osteoporo*s*. 2019;14(1):29. 10.1007/s11657-019-0583-4 30826896

[23] Chastin SF , Mandrichenko O, Helbostadt JL, Skelton DA. Associations between objectively-measured sedentary behaviour and physical activity with bone mineral density in adults and older adults, the NHANES study. Bon*e*. 2014 Jul;64:254–262. 10.1016/j.bone.2014.04.009 24735973

[24] Johansson J , Nordstrom A, Nordstrom P. Objectively measured physical activity is associated with parameters of bone in 70-year-old men and women. Bon*e*. 2015 Dec;81:72–79. 10.1016/j.bone.2015.07.001 26151120

[25] Rodriguez-Gomez I , Manas A, Losa-Reyna J, et al. Associations between sedentary time, physical activity and bone health among older people using compositional data analysis. PLoS On*e*. 2018;13(10):e0206013. 10.1371/journal.pone.0206013 30346973 PMC6197664

[26] Jacobsen BK , Eggen AE, Mathiesen EB, Wilsgaard T, Njolstad I. Cohort profile: the Tromso study. Int J Epidemio*l*. 2012;41(4):961–967. 10.1093/ije/dyr049 21422063 PMC3429870

[27] Statistics Norway (SSB) . Populatio*n*. 2019: [updated 16 November 2023]. Available from: https://www.ssb.no/en/befolkning/statistikker/folkemengde/aar-per-1-januar. Accessed 11 February 2024.

[28] Hopstock LA , Grimsgaard S, Johansen H, Kanstad K, Wilsgaard T, Eggen AE. The seventh survey of the Tromso study (Tromso7) 2015-2016: study design, data collection, attendance, and prevalence of risk factors and disease in a multipurpose population-based health survey. Scand J Public Healt*h*. 2022;50(7):919–929. 10.1177/14034948221092294 35509230 PMC9578102

[29] Sagelv EH , Hopstock LA, Johansson J, et al. Criterion validity of two physical activity and one sedentary time questionnaire against accelerometry in a large cohort of adults and older adults. BMJ Open Sport Exerc Me*d*. 2020;6(1):e000661. 10.1136/bmjsem-2019-000661 PMC7047487 32153981

[30] Sagelv EH , Ekelund U, Pedersen S, et al. Physical activity levels in adults and elderly from triaxial and uniaxial accelerometry. PLoS *One*. 2019;14(12):e0225670. 10.1371/journal.pone.0225670 31794552 PMC6890242

[31] Omsland TK , Emaus N, Gjesdal CG, et al. In vivo and in vitro comparison of densitometers in the NOREPOS study. J Clin Densito*m*. 2008;11(2):276–282. 10.1016/j.jocd.2007.10.001 18158262

[32] Rao AD , Reddy S, Rao DS. Is there a difference between right and left femoral bone density? J Clin Densito*m*. 2000;3(1):57–61. 10.1385/JCD:3:1:057 10745302

[33] Cauley JA , Giangregorio L. Physical activity and skeletal health in adults. Lancet Diabetes Endocrino*l*. 2020;8(2):150–162. 10.1016/S2213-8587(19)30351-1 31759956

[34] Office of the Surgeon General (US). Bone Health and Osteoporosis: A Report of the Surgeon General. Reports of the Surgeon General. Rockville (MD); 2004. 20945569

[35] World Health Organization (WHO). WHO Guidelines on Physical Activity and Sedentary Behaviour. WHO Guidelines Approved by the Guidelines Review Committe*e*. Geneva; 2020. 33369898

[36] Hecht A , Ma S, Porszasz J, Casaburi R, Network CCR. Methodology for using long-term accelerometry monitoring to describe daily activity patterns in COPD. COP*D*. 2009;6(2):121–129. 10.1080/15412550902755044 19378225 PMC2862250

[37] Sasaki JE , John D, Freedson PS. Validation and comparison of ActiGraph activity monitors. J Sci Med Spor*t*. 2011;14(5):411–416. 10.1016/j.jsams.2011.04.003 21616714

[38] Kozey-Keadle S , Libertine A, Lyden K, Staudenmayer J, Freedson PS. Validation of wearable monitors for assessing sedentary behavior. Med Sci Sports Exer*c*. 2011;43(8):1561–1567. 10.1249/MSS.0b013e31820ce174 21233777

[39] Mohebbi R , Shojaa M, Kohl M, et al. Exercise training and bone mineral density in postmenopausal women: an updated systematic review and meta-analysis of intervention studies with emphasis on potential moderators. Osteoporos In*t*. 2023;34(7):1145–1178. 10.1007/s00198-023-06682-1 36749350 PMC10282053

[40] Soares WJS , Lopes AD, Nogueira E, Candido V, de Moraes SA, Perracini MR. Physical activity level and risk of falling in community-dwelling older adults: systematic review and meta-analysis. J Aging Phys Ac*t*. 2019;27(1):34–43. 10.1123/japa.2017-0413 29543113

[41] Szulc P , Munoz F, Duboeuf F, Marchand F, Delmas PD. Bone mineral density predicts osteoporotic fractures in elderly men: the MINOS study. Osteoporos In*t*. 2005;16(10):1184–1192. 10.1007/s00198-005-1970-9 16096713

[42] Weaver CM , Gordon CM, Janz KF, et al. The National Osteoporosis Foundation's position statement on peak bone mass development and lifestyle factors: a systematic review and implementation recommendations. Osteoporos In*t*. 2016;27(4):1281–1386. 10.1007/s00198-015-3440-3 26856587 PMC4791473

[43] Khan K , McKay HA, Haapasalo H, et al. Does childhood and adolescence provide a unique opportunity for exercise to strengthen the skeleton? J Sci Med Spor*t*. 2000;3(2):150–164. 10.1016/S1440-2440(00)80077-8 11104307

[44] Robling AG , Hinant FM, Burr DB, Turner CH. Improved bone structure and strength after long-term mechanical loading is greatest if loading is separated into short bouts. J Bone Miner Re*s*. 2002;17(8):1545–1554. 10.1359/jbmr.2002.17.8.1545 12162508

[45] Rubin CT , Bain SD, McLeod KJ. Suppression of the osteogenic response in the aging skeleton. Calcif Tissue In*t*. 1992;50(4):306–313. 10.1007/BF00301627 1571841

[46] Olmedillas H , Gonzalez-Aguero A, Moreno LA, Casajus JA, Vicente-Rodriguez G. Cycling and bone health: a systematic review. BMC Me*d*. 2012;10(1):168. 10.1186/1741-7015-10-168 23256921 PMC3554602

[47] Harrison F , Atkin AJ, van Sluijs EMF, Jones AP. Seasonality in swimming and cycling: exploring a limitation of accelerometer based studies. Prev Med Re*p*. 2017 Sep;7:16–19. 10.1016/j.pmedr.2017.04.006 28593117 PMC5447377

